# Media formulation using statistical methodology to enhance α-amylase production for green synthesis of Au-NPs by *Bacillus subtilis* VSP4 under solid-state fermentation

**DOI:** 10.3389/fbioe.2025.1569902

**Published:** 2025-06-03

**Authors:** Vimalkumar S. Prajapati, Vaibhavkumar N. Mehta, Swati K. Patel

**Affiliations:** ^1^ Division of Microbial and Environmental Biotechnology, ASPEE SHAKILAM Biotechnology Institute (ASBI), Navsari Agricultural University, Surat, Gujarat, India; ^2^ Center of Excellent in Nano Agri-Biotechnology, ASPEE SHAKILAM Biotechnology Institute (ASBI), Navsari Agricultural University, Surat, Gujarat, India; ^3^ Division of Plant Biotechnology, ASPEE SHAKILAM Biotechnology Institute (ASBI), Navsari Agricultural University, Surat, Gujarat, India

**Keywords:** α-amylase production, gold nanoparticles, Plackett–Burman design, central composite design, response surface methodology, solid-state fermentation

## Abstract

In recent years, gold nanoparticles (Au-NPs) have garnered popularity for their remarkable and promising applications in various areas. Here, we report the synthesis of Au-NPs using extracellular amylase produced by *Bacillus subtilis* VSP4 under solid-state fermentation (SSF) through the reduction of AuCl_4_ with retention of enzymatic activity in the complex. Accordingly, *B. subtilis* VSP4 was exploited to enhance α-amylase production under SSF using the Plackett–Burman design, followed by the central composite design (CCD) of response surface methodology (RSM). According to our analysis, the most significant components in the medium are starch, yeast extract, and CaCl_2_ (significance >95%, ANOVA), which prominently enhance enzyme production. The optimum levels of these three selected variables were evaluated using CCD-RSM (20 runs), and it was confirmed that 0.05 g of starch, 0.1 g of yeast extract, and 5 mM of CaCl_2_ per 5 g of wheat bran under SSF produced the maximum α-amylase yield (169.72 U/gds). The *F*-value of the quadratic model (18.36) implies that the model is significant, while the *F*-value of the lack of fit (3.17) indicates that the lack of fit is not significant, meaning that the model has good fit. The coefficient of variance was found to be 0.369, which denotes that the experiments performed herein are reliable (R^2^ = 0.94) (multiple correlation coefficient), and the standard deviation for the quadratic model was found to be 4.72. We also performed separate validation experiments to confirm the adequacy of the quadratic model. The present work highlights α-amylase production by *B. subtilis* VSP4 under SSF, which was prominently enhanced by adopting a statistical experimental design, leading to the formation of Au-NPs of average size 5.17 ± 0.85 nm showing a surface plasmon resonance peak at 528 nm.

## Graphical Abstract

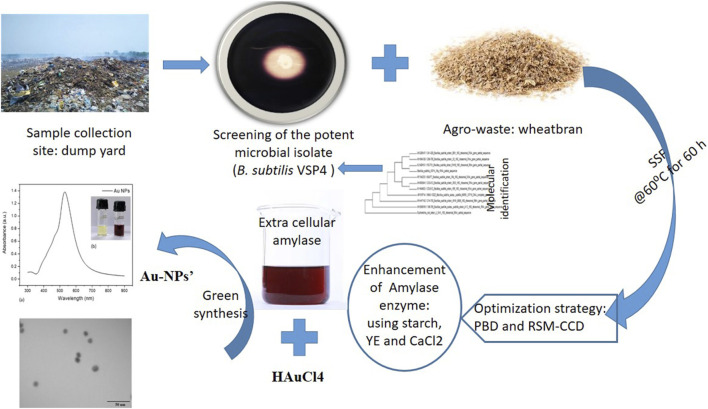

## 1 Introduction

Nanotechnology has gained increasing attention in recent years owing to its various applications in diverse fields. Gold nanoparticles (Au-NPs) are among the most popular developments of nanotechnology and are widely used in therapeutics for anti-angiogenesis (Mukherjee et al., 2005), as antimalarial and anti-arthritic agents (Tsai et al., 2007), and as agents in biohydrogen production (Zhang and Shen, 2007) etc. owing to their extraordinary physicochemical properties. Hence, there is a great need for developing biocompatible, high-yield, low-cost, non-toxic, and environment-friendly processes for the synthesis of metallic nanoparticles through biological approaches, which are more preferrable than various physical and chemical methodologies (Kalishwaralal et al., 2010; Soto et al., 2021). Amylase is an essential enzyme that plays a fundamental role in the field of biotechnology (Gopinath et al., 2017). Optimized media for extracellular α-amylase production from *Bacillus licheniformis* have been adopted for the green synthesis of Au-NPs. Biomacromolecules have also been previously employed to synthesize metal oxides, with reports indicating that proline residues in the alpha enzyme play crucial roles in the reduction process, making it a more effective synthesizer of nanoparticles (Kalishwaralal et al., 2010).

Amylases are increasingly being sourced from microorganisms (primarily bacteria and fungi), plants, and animals. However, microorganisms have garnered the most attention owing to the distinct characteristics of the amylases produced by them. These amylases are thermally more stable, yield varied sugar profiles, and are better suited to meet industrial demands than those derived from plants and animals. Among amylases, α-amylases [EC 3.2.1.1] are enzymes that can randomly cleave the 1,4-α-D-glycosidic linkages between adjacent glucose molecules in linear polysaccharides, such as starch, glycogen, and oligosaccharides (Kizhakedathil and Chandrasekaran, 2018). Amylases constitute approximately 25% of the global enzyme market (Burhan et al., 2003), and this biocatalyst has mostly replaced chemical hydrolysis of starch in various industries (Pandey et al., 2000a). Several bacteria have been found to be capable of producing tremendous amounts of α-amylase for industrial applications, including *Bacillus amyloliquefaciens* (Gangadharan et al., 2011; Prajapati et al., 2015; Gangadharan et al., 2008), *B. licheniformis* (Karataş et al., 2012; Huang et al., 2019), and *Bacillus stearothermophilus* (Wang et al., 2019; Xie et al., 2019). Thermostable α-amylases have broad profitable applications in various starch processing industries, brewing and sugar production (Lévêque et al., 2000), textile industries (Hendriksen et al., 1999; Sivaramakrishnan et al., 2006), detergent manufacturing processes, as well as biological formulation of various nanostructures. The majority of microbial amylases are predominantly produced by submerged fermentation and solid-state fermentation (SSF) processes, but SSF is considered to be the superior technique for enzyme production owing to its various advantages like high productivity, simplicity, low production cost, low energy requirement, better product recovery, and reduced catabolite repression (Pandey, 1992; Pandey et al., 2000b; Pandey, 2003; Singh et al., 2020).

Implementing statistical methodologies to optimize the media used for enzyme production has several advantages over conventional methods as it reduces experimentation time, allows evaluation of many parameters in just a few statistical blocks, and decreases the cost of the media, which directly justify the fermentation economics and provide the best outputs that can be validated via further experiments. In the present investigation, the statistical method is used to formulate the fermentation media for the production of a potentially thermostable α-amylase from *B. subtilis* VSP4 under SSF by employing the Plackett–Burman design (PBD) and response surface methodology (RSM)-based central composite design (CCD) as an effective media optimization approach. The amylase produced by *B. subtilis* using the optimized media was used to test the synthesis of nanoparticles, which revealed that this process allowed enhanced and rapid synthesis of Au-NPs. To the best of our knowledge, the interactions of yeast extract and CaCl_2_ having affirmative effects on α-amylase production under SSF is being reported for the first time herein.

## 2 Materials and methods

### 2.1 Strain isolation, identification, and inoculum preparation

The screening and isolation were conducted on the basis of the ability to produce extracellular amylases on starch agar plates. Accordingly, a clear or colorless zone will appear around the bacterial growth, signifying that the amylase has hydrolyzed (broken down) the starch. Once iodine is added to the plate, the unaffected starch will turn blue-black in color. Soil samples were collected from the Khajod dump site in Surat, Gujarat, India. Wheat bran was used for the SSF and purchased from a local market in Surat. The culture was maintained at 4°C on Bushnell Hass agar (BHA) medium (0.2 g/L of MgSO_4_, 0.02 g/L of CaCl_2_, 1.00 g/L of KH_2_PO_4_, 1.00 g/L of K_2_HPO_4_, 1.00 g/L of NH_4_NO_3_, and 0.05 g/L of FeCl_3_ at pH 7.0) slants containing 1% starch. The bacterial isolate VSP4 was identified via amplification of the 16S rDNA gene using the universal set of primers (forward: 5′ AGA​GTT​TGA​TCC​TGG​CTC​AG 3′, reverse: 5′ GGT​TAC​CTT​GTT​ACA​GCT​T 3′). The phylogenetic relationship of the isolate VSP4 was governed by comparing the sequence data using blast tool with the existing sequences available in the GeneBank database of the National Centre for Biotechnology Information (NCBI), U.S. National Library of Medicine, Bethesda, MD, United States. The inoculum was prepared in 50 mL of nutrient broth (NB) inoculated with 24-h-grown microbial culture at 37°C under shaking, and an appropriate aliquot was added to the experimental flasks to obtain the desired optical density (OD) of ∼8 × 10^7^ CFU/mL (Prajapati et al., 2015).

### 2.2 Enzyme production, extraction, and assay

Enzyme production was carried out in 250-mL Erlenmeyer flasks containing 5 g of wheat bran moist with 10 mL of tap water. The production media were autoclaved at 121°C and 15 psi of pressure for 25 min. These conditions are applied to sterilize the SSF medium to ensure removal of any microbial contaminants prior to inoculating with the target microorganism. All fermentation flasks were inoculated with 1 OD unit of the microbial culture and incubated at 60°C, followed by harvesting at 60-h intervals. Enzyme extraction was carried out using 40 mL of phosphate buffer (0.05 M; pH 8.0) on a rotary shaker at 150 rpm and 25°C for 30 min. The entire contents were then sieved through a muslin cloth, followed by centrifugation at 9,000 rpm for 15 min. The obtained supernatant was used to measure the enzyme yield and represented in terms of units/gram dry substrate (U/gds) (Prajapati et al., 2013; Miller, 1959). Then, corn starch was hydrolyzed using the obtained enzyme, and the enzymatic products were assessed using thin layer chromatography (TLC). The TLC was conducted using a solvent mixture of butanol, acetic acid, and water in a 4:1:1 ratio. A standard sugar mixture (such as glucose and maltose) was spotted on the plate for comparison. After the plate was developed, it was sprayed with aniline diphenylamine (ADA), which reacts with the sugars to produce colored spots when heated at 100°C for 5 min. We note that the enzymatic hydrolysis revealed the formation of maltose as a major end product, which confirms the enzyme as an α-amylase (Prajapati et al., 2015).

### 2.3 Implementation of statistical strategy to enhance α-amylase production

The PBD approach (Prajapati et al., 2013; Plackett and Burman, 1946) and CCD-RSM-based experiments were performed according to the design matrix shown in Tables 1, 2, respectively. The initial experiment to formulate the media for maximizing α-amylase production by *B. subtilis* VSP4 involved the one factor at a time (OFAT) methodology. The OFAT experiment indicated that significant α-amylase production by *B. subtilis* under SSF occurred after 60 h of incubation at 60°C and an intermediate pH range of 9–10. The addition of starch and yeast extract led to a notable increase in enzyme production. Various chemical (effects of carbon and nitrogen sources) and physical (effects of incubation time, temperature, and pH) process parameters were considered to narrow the list of parameters for further optimization. The PBD matrix (12 runs) helped in identifying the significant variables for α-amylase production, and the chosen variables and their concentration ranges selected for the study are listed in Table 3. ANOVA was performed on the obtained data to assess the main effects of the components, the experimental standard errors, and percentage significance of each selected variable (Prajapati et al., 2013).

**TABLE 1:** Plackett–Burman design (PBD) generated by fractional rotation of the full factorial design, where X1–X7 are independent variables and D1–D4 are dummy variables.

Run no.	X1	X2	X3	X4	X5	X6	X7	X8	X9	X10	X11	Amylase production (U/gds)
	Maltose	Starch	Yeast extract	Casein AH	pH	MgSO_4_·7H_2_O	CaCl_2_	D1	D2	D3	D4	
1	High	High	Low	High	High	High	Low	Low	Low	High	Low	21.34
2	Low	High	High	Low	High	High	High	Low	Low	Low	High	07.87
3	High	low	High	High	Low	High	High	High	Low	Low	Low	04.22
4	Low	High	Low	High	High	Low	High	High	High	Low	Low	11.47
5	Low	Low	High	Low	High	High	Low	High	High	High	Low	09.41
6	Low	Low	Low	High	Low	High	High	Low	High	High	High	07.32
7	High	low	Low	Low	High	Low	High	High	low	High	High	04.22
8	High	High	Low	Low	Low	High	low	High	High	Low	High	14.41
9	High	High	High	Low	Low	Low	High	Low	High	High	Low	07.16
10	Low	High	High	High	Low	Low	Low	High	Low	High	High	11.57
11	High	Low	High	High	High	Low	Low	low	High	Low	High	03.53
12	Low	Low	Low	Low	Low	Low	Low	Low	Low	Low	Low	12.65

**TABLE 2:** Central composite design based on response surface methodology (CCD-RSM) with actual and coded values of the components and their responses.

Std	Run	Factor 1	Factor 2	Factor 3	Response 1	
		A: Starch	B: Yeast extract	C: CaCl_2_	Amylase production (U/gds)	
		g	g	mM	Actual	Predicted
1	14	0.05	0.01	0.5	159.91	164.38
2	11	0.5	0.01	0.5	163.18	155.87
3	9	0.05	0.1	0.5	126.67	123.7
4	7	0.5	0.1	0.5	107.68	109.05
5	12	0.05	0.01	5	116.02	120.23
6	8	0.5	0.01	5	121.09	123.7
7	1	0.05	0.1	5	108.19	105.36
8	16	0.5	0.1	5	120.46	122.06
9	18	−0.10	0.055	2.75	125.53	126.12
10	4	0.65	0.055	2.75	124.9	123.7
11	13	0.275	−0.02	2.75	120.22	117.94
12	17	0.275	0.13	2.75	133.12	138.15
13	5	0.275	0.055	−1.03	120.73	119.67
14	2	0.275	0.055	6.53	127.69	126.9
15	20	0.275	0.055	2.75	118.18	123.7
16	6	0.275	0.055	2.75	133.51	136.49
17	3	0.275	0.055	2.75	133.18	131.14
18	15	0.275	0.055	2.75	144.52	140.04
19	19	0.275	0.055	2.75	125.17	123.7
20	10	0.275	0.055	2.75	125.65	123.7

**TABLE 3:** Components of the production medium and their variables used in the PBD for amylase production.

Variables	Medium components	(+) value	(−) value
X1	Maltose	0.3 g	0.02 g
X2	Starch	0.3 g	0.02 g
X3	Yeast extract	0.3 g	0.01 g
X4	Casein AH	0.3 g	0.01 g
X5	pH	9	10
X6	MgSO_4_·7H_2_O	10 mM	1 mM
X7	CaCl_2_	10 mM	1 mM

PBD aided screening of the three major components of the medium, namely, starch, yeast extract, and calcium chloride, for α-amylase production; the levels of these three parameters were further optimized using CCD-RSM. All three variables were tested at five different levels (−α, −1, 0, +1, and +α), and their concentration ranges are depicted in Table 4 on the basis of previous experiments. The complete experimental plan comprises 20 runs of the design matrix with actual and coded values, and the corresponding responses are presented in Table 2. Statistical analysis was performed to obtain the quadratic model followed by assessment of the response surface plots. To assess the competence of the optimized value of each component, an additional validation experiment was carried out to confirm α-amylase production (Prajapati et al., 2015). The statistical software Design Expert version 10.0 (Stat-Ease Inc., Minneapolis, Minnesota, United States) was used for the data analyses and validation experiments.

**TABLE 4:** Levels of the selected variables for CCD-RSM.

Variables	Components	Range	Variable levels				
			−α	−1	0	+1	+α
X1: A	Starch	0.05–0.50	−0.10	0.05	0.275	0.50	0.65
X2: B	Yeast extract	0.01–0.10	−0.02	0.01	0.055	0.10	0.13
X3: C	CaCl_2_	0.5–5.00	−1.03	0.50	2.750	5.00	6.53

### 2.4 Biosynthesis and evaluation of the Au-NPs

Green synthesis of the Au-NPs was carried out according to a method reported previously (Kalishwaralal et al., 2010, 2009) with some modifications. The general cultivation media, NB, and RSM-optimized production media (SSF) for enhanced amylase yield were inoculated with 1.0 OD of *B. subtilis* VSP4. The NB-inoculated flask was incubated at 37°C and 220 rpm for 24 h, while the RSM-optimized flask was incubated at 60°C and harvested after 60 h. At the end of the incubation period, the NB was subjected to centrifugation at 8,000×*g*, and the supernatant was used to synthesize the Au-NPs; similarly, the SSF flask was subjected to enzyme extraction, as stated earlier. Two Erlenmeyer flasks, one containing the supernatant collected from the NB flasks and the second containing the supernatant with the extracted enzyme using HAuCl_4_ (Merck, Germany, 99.9% pure) at a concentration of 50 mM, were incubated for 24 h. After 24 h of incubation, the absorption spectra of the samples were recorded on a Lab India 3000 spectrophotometer (India).

### 2.5 Characterization of the Au-NPs

The morphology of the biosynthesized Au-NPs was characterized using transmission electron microscopy (TEM). The TEM samples were prepared on a copper grid coated with a carbon film and analyzed using Tecnai 20 (Phillips, Holland) at an accelerating voltage of 100 kV. The UV–visible spectra were measured using a Lab India 3000 spectrophotometer (India) at room temperature.

## 3 Results

**FIGURE 1:**
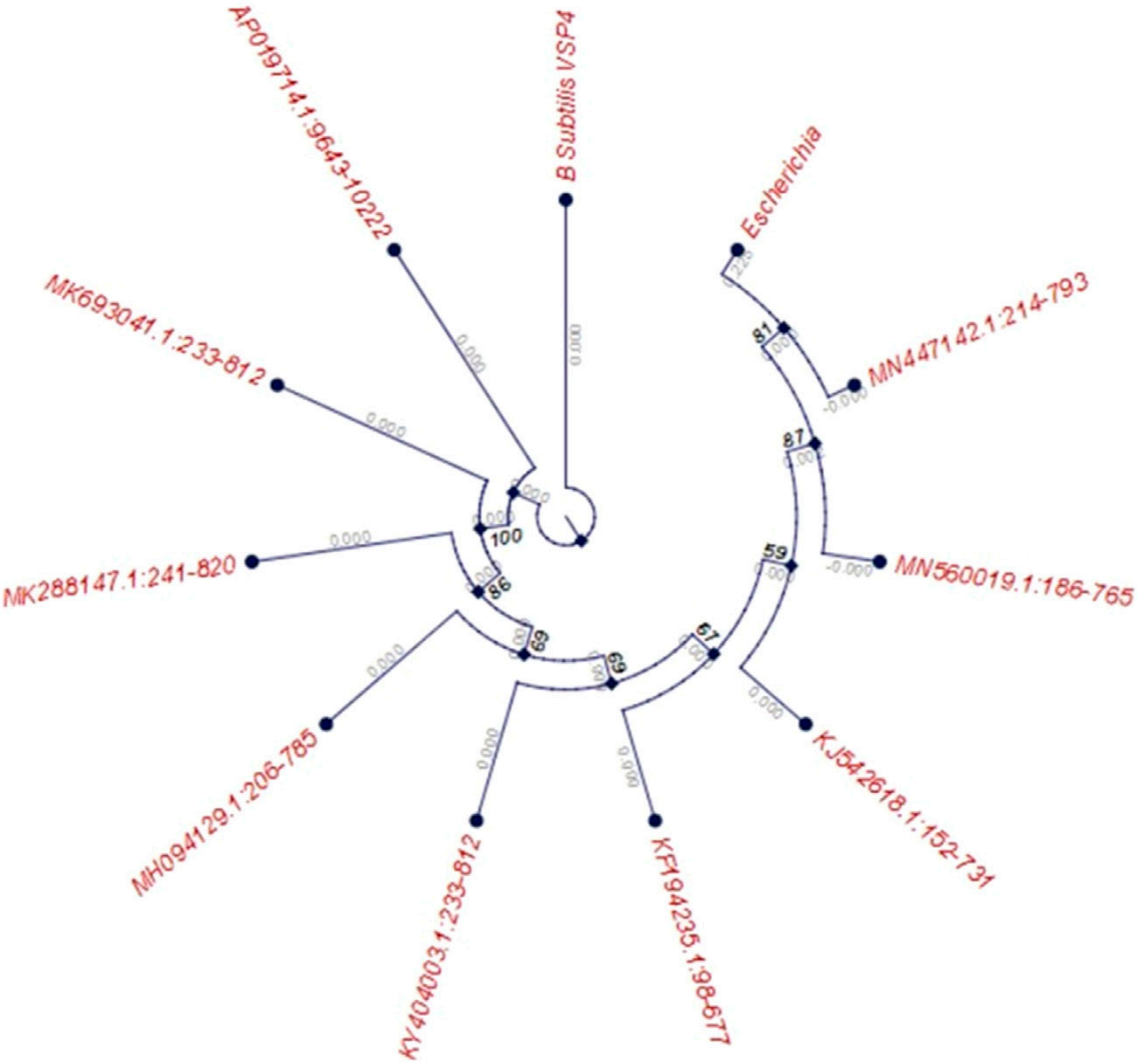
Phylogenetic tree of the screened isolate *Bacillus subtilis* VSP4 based on 16S rRNA gene sequencing (bootstrap consensus tree based on 500 replicates represents the evolutionary history of the taxa, with the branches collapsed if reproduced in fewer than 50% of the replicates).

Around 25 microbial strains were isolated from the collected soil samples and screened on starch agar plates for amylase production. Of these 25 strains, isolate VSP4 was chosen for further optimization as it exhibited better amylolytic activity on the starch agar plate, as determined by the ratio of diameter of the amylolytic zone to the growth diameter. The isolate VSP4 was identified as *B. subtilis* VSP4 through polymerase chain reaction (PCR) amplification of the 16s rRNA gene using the universal set of primers followed by sequencing of the PCR product. The obtained sequence has been deposited in the NCBI database (GenBank accession no. MN960687; https://www.ncbi.nlm.nih.Gov/nuccore/MN960687.1). The evolutionary history of the strain was inferred using the neighbor-joining method. Evolutionary analyses were conducted in MEGA6, and the phylogeny tree of the screened isolate *B. subtilis* VSP4 is shown in Figure 1.

### 3.1 Selection of the influential process parameters

After performing the OFAT experiment, the PBD approach was used to shortlist the significant parameters for enhanced amylase production using *B. subtilis* VSP4. The ANOVA results for the PBD matrix (12 runs) with respect to α-amylase production under SSF using *B. subtilis* VSP4 are presented in Table 5. The significant constituents of the media, i.e., starch, yeast extract, and CaCl_2_, were identified using the PBD and further studied for level optimization to assess the interaction effects using CCD-RSM for enhanced α-amylase production.

**TABLE 5:** Statistical analysis of the components for amylase production based on PBD.

Components	Effect	Std. Error	*t*-value	*p*-value	% confidence
Maltose	−0.903	1.7	−0.531	0.623	37.659
Starch	5.413	1.7	3.184	0.033	96.659
Yeast extract	−4.609	1.7	−2.711	0.053	94.652
Casein AH	0.622	1.7	0.366	0.733	26.702
pH	0.086	1.7	0.051	0.962	3.795
MgSO_4_·7H_2_O	2.330	1.7	1.371	0.242	75.763
CaCl_2_	−5.110	1.7	−3.006	0.040	96.029

### 3.2 Evaluation of the optimal process parameters using CCD-RSM

CCD-RSM was implemented to understand the interactions among starch, yeast extract, and CaCl_2_ to obtain the maximum amylase yield. These experiments were focused on obtaining a quadratic model with 2^3^ trials and included 20 experiments at different combinations of the selected components, as presented in the design matrix (Table 2) along with the predicted and observed responses (α-amylase production in U/gds) of the individual runs. The ANOVA results of the quadratic model (CCD-RSM) for the observed enzyme yields under SSF by *B. subtilis* VSP4 are given in Table 6. The *F*-values for the quadratic model and lack of fit were found to be 18.36 and 3.17, respectively. Thus, the yield of α-amylase by *B. subtilis* VSP4 may be best anticipated by the regression expression shown in Equation 1 and obtained from ANOVA as a function of the three selected media components:

$$\text{Amylase production }\left(Y\right)=\left(123.69\right)\;–\;\left(9.21\times A\right)+\left(3.411\times B\right)+\left(10.59\times C\right)\;‐\;\left(2.95\times A\times B\right)\;–\left(1.78\times A\times C\right)+\left(6.75\times B\times C\right)+\left(0.03\times A^{2}\right)+\left(0.60\times B^{2}\right)+\left(5.07\times C^{2}\right),$$ (1)

where Y is the α-amylase production (U/gds), A is the starch concentration (w/w), B is the concentration of yeast extract (w/w), and C is the concentration of calcium chloride (mM).

**TABLE 6:** ANOVA of the quadratic model (CCD-RSM) for enzyme yield.

Source	Sum of squares	df	Mean square	*F*-value	*p*-value Prob > F	Relevance
Model	3,682.766	9	409.1962	18.36248	4.36E-05	Significant
A: Starch	1,159.308	1	1,159.308	52.02336	2.88E-05	Significant
B: Yeast extract	158.9291	1	158.9291	7.131865	0.02347	Significant
C: CaCl_2_	1,532.803	1	1,532.803	68.78379	8.58E-06	Significant
AB	69.67901	1	69.67901	3.126811	0.107452	
AC	25.38281	1	25.38281	1.139041	0.310943	
BC	364.9051	1	364.9051	16.37494	0.002336	Significant
A^2^	1.301761	1	1.301761	0.058416	0.813902	
B^2^	5.237231	1	5.237231	0.235018	0.638271	
C^2^	370.9552	1	370.9552	16.64643	0.002214	Significant
Residual	222.8437	10	22.28437			
Lack of fit	169.3855	5	33.8771	3.16856	0.115666	Not Significant
Pure error	53.4582	5	10.69164			
Cor total	3,905.609	19				

**FIGURE 2:**
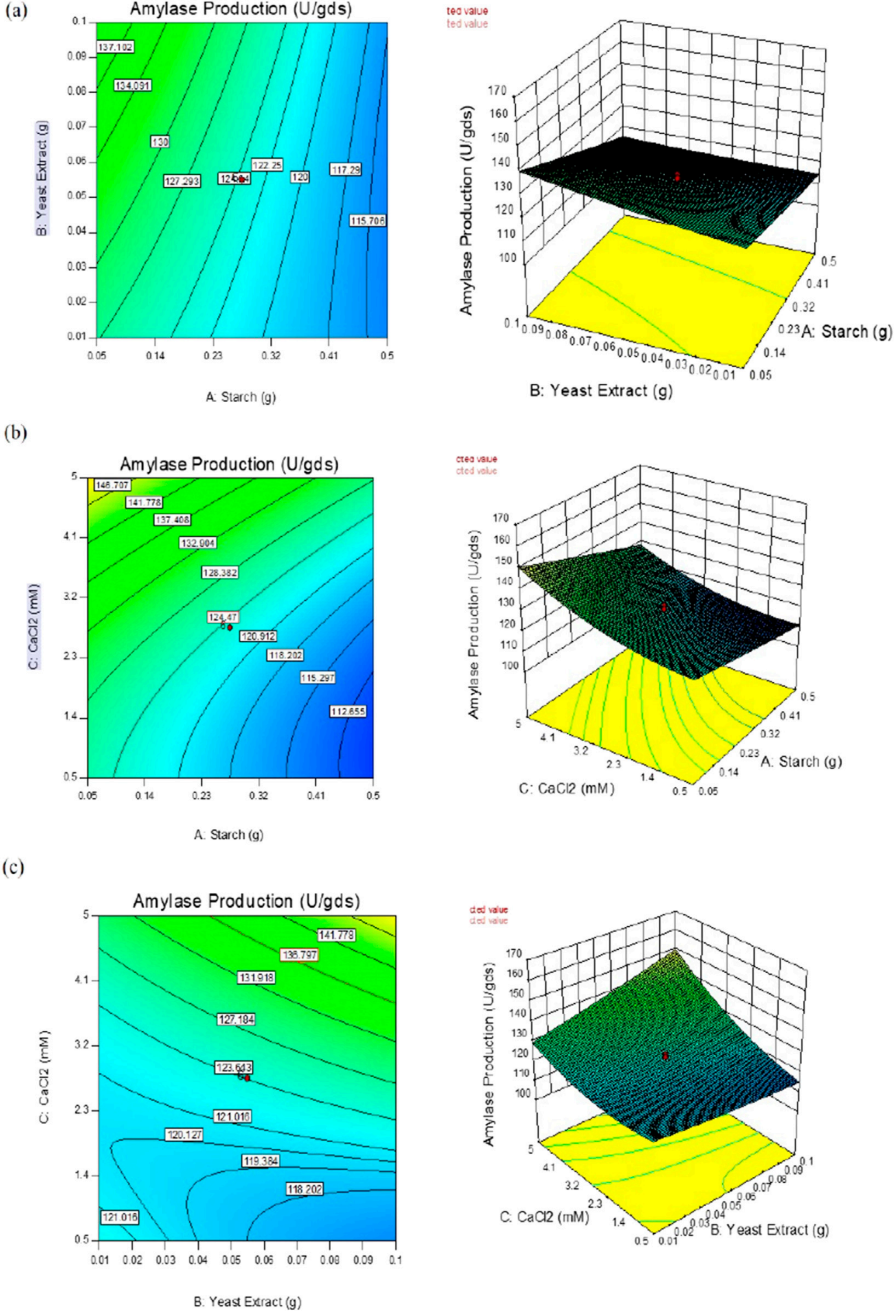
Response surface plots of α-amylase production by *B. subtilis* VSP4 under solid-state fermentation (SSF) showing the interaction effects between concentrations of **(a)** starch and yeast extract; **(b)** starch and CaCl_2_; **(c)** yeast extract and CaCl_2_.

The interactive effects of the chosen variables on enzyme yield (U/gds) were investigated against any two autonomous variables by fixing the concentration of the third variable. These model graphs are depicted in Figure 2, and the plots can be used to envisage the ideal concentrations of all selected variables to enhance enzyme yield under SSF.

### 3.3 Validation of the quadratic model

Validation experiments were conducted by choosing the levels of all components predicted by the response surface model using the numerical optimization method in Design Expert v.10.0. The ideal concentrations of the selected variables were 0.05 g for starch, 0.1 g for yeast extract, and 5 mM for calcium chloride per 5 g of wheat bran for the SSF experiment with *B. subtilis* VSP4, which resulted in an enzyme yield of 164.38 U/gds as per the model prediction. Additional experiments were performed in triplicate with the boosted levels of each of the components in the SSF medium at pH 10.0 and fed with 1 OD of *B. subtilis* VSP4 incubated at 60°C, followed by harvesting after 60 h to authenticate the model prediction. The enzyme yield was found to be 169.72 U/gds, which is comparable to the yield predicted by the model.

### 3.4 Biosynthesis and characterization of Au-NPs

**FIGURE 3:**
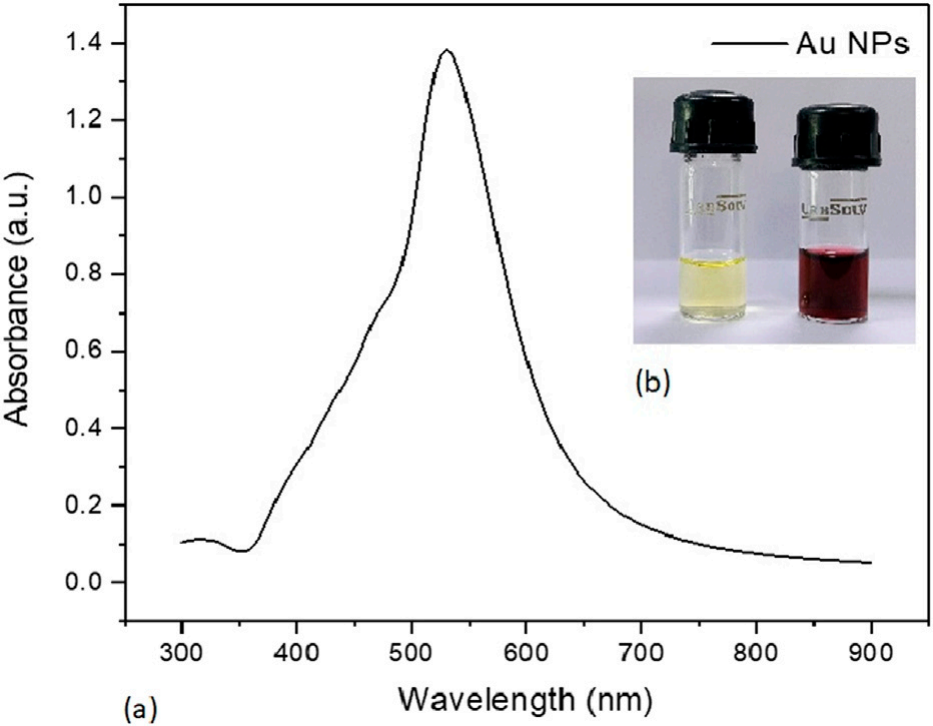
**(a)** UV–visible spectrum of the biosynthesized gold nanoparticles. **(b)** Color changes observed for α-amylase-mediated biosynthesis of gold nanoparticles (inset).

**FIGURE 4:**
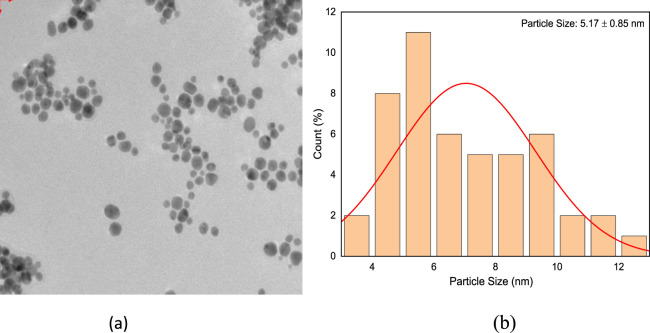
**(a)** Transmission electron micrograph of the biosynthesized gold nanoparticles having an average size of 5.17 ± 0.85 nm and **(b)** histogram showing the size distribution of the gold nanoparticles.

The culture supernatant of *B. subtilis* VSP4 allowed efficient reduction of the HAuCl_4_ ions to Au-NPs. It was visually observed that the RSM-optimized production medium incubated with 50 mM of HAuCl_4_ underwent a color change from whitish yellow to red, whereas no color change was observed in the culture supernatant without HAuCl_4_ (Figure 3b inset). The UV–visible spectrum of the biosynthesized Au-NPs using the supernatant containing amylase shows the characteristic surface plasmon resonance peak at 528 nm, confirming the formation of Au-NPs (Figure 3a). Furthermore, the morphology of the biosynthesized Au-NPs was investigated using TEM, which revealed monodispersed spherical particles of average size 5.17 ± 0.85 nm when prepared using α-amylase from *B. subtilis* VSP4 (Figures 4a,b).

## 4 Discussion

An intensive statistical strategy was adopted to shortlist the most effective media components and determine their optimal concentrations to accomplish the best possible α-amylase production on wheat bran substrate using *B. subtilis* VSP4. OFAT experiments were conducted to select the list of parameters to be analyzed further using the statistical methodology. The components were initially screened using the PBD approach to understand their effects on enzyme production, of which a few important components were selected for optimization. As per the ANOVA results, only those media constituents showing significance levels of 95% or above were selected (Plackett and Burman, 1946). In our experiments, we observed that starch, yeast extract, and CaCl_2_ presented confidence levels >95% and were considered to be significant while the remaining components showed confidence levels <95% and were deemed insignificant for the study. The presence of starch in the fermentation medium is important as it acts as an inducer that is necessary for product fermentation. Yeast extract is known to be one of the cheapest sources of nitrogen and provides various amino acids, vitamins, and minerals that support microbial growth during the lag phase, leading to product formation. Calcium chloride plays a vital role in maintaining the integrity of α-amylase and hence its stability; it helps by holding the protein structure in the correct configuration and resisting thermal inactivation.

The CCD approach was used to study the interactions between and among the selected media components as well as help determine their optimal concentrations to achieve the best enzyme yield (Prajapati et al., 2015; Jegatheesan and Eyini, 2015). The *F*-test analysis was used to assess the significance of the quadratic model in response to the experiments to achieve the highest possible α-amylase yield (Prajapati et al., 2013, 2014). Normally, a larger *F*-value is obtained for a model in the presence of noise, but the occurrence rate of such a case is only 0.01%. In the present study, the *F*-value for the quadratic model was 18.36, and values less than 0.05 were used to identify the significant model terms. Table 6 shows that model terms A, B, C, BC, and C^2^ are highly significant, while lack of fit is not significant as it has an *F*-value of 3.17. The non-significant lack of fit implies that the proposed model has good fit. An *R* ^2^ value (0.94) (multiple correlation coefficient) close to unity indicates better correlation between the observed and predicted values (Kizhakedathil and Chandrasekaran, 2018; Pujari and Chandra, 2000), while the standard deviation for the model was found to be 4.72. The experiments were further compared by the degree of consistency, as indicated by the coefficient of variation (CV). For the present study, we obtained a CV of 3.69%, which proves that the experiments are consistent, while large CV values indicate poorer reliability of the experiments (Singh et al., 2020). The signal-to-noise ratio of the model is represented by adequate precision; for an appropriate quadratic model, the desired ratio should be greater than 4. In the present study, this ratio was found to be 17.68, indicating an adequate signal; this also suggests that the model is valid and can be effectively used to navigate the design space (Raol et al., 2014).

**FIGURE 5:**
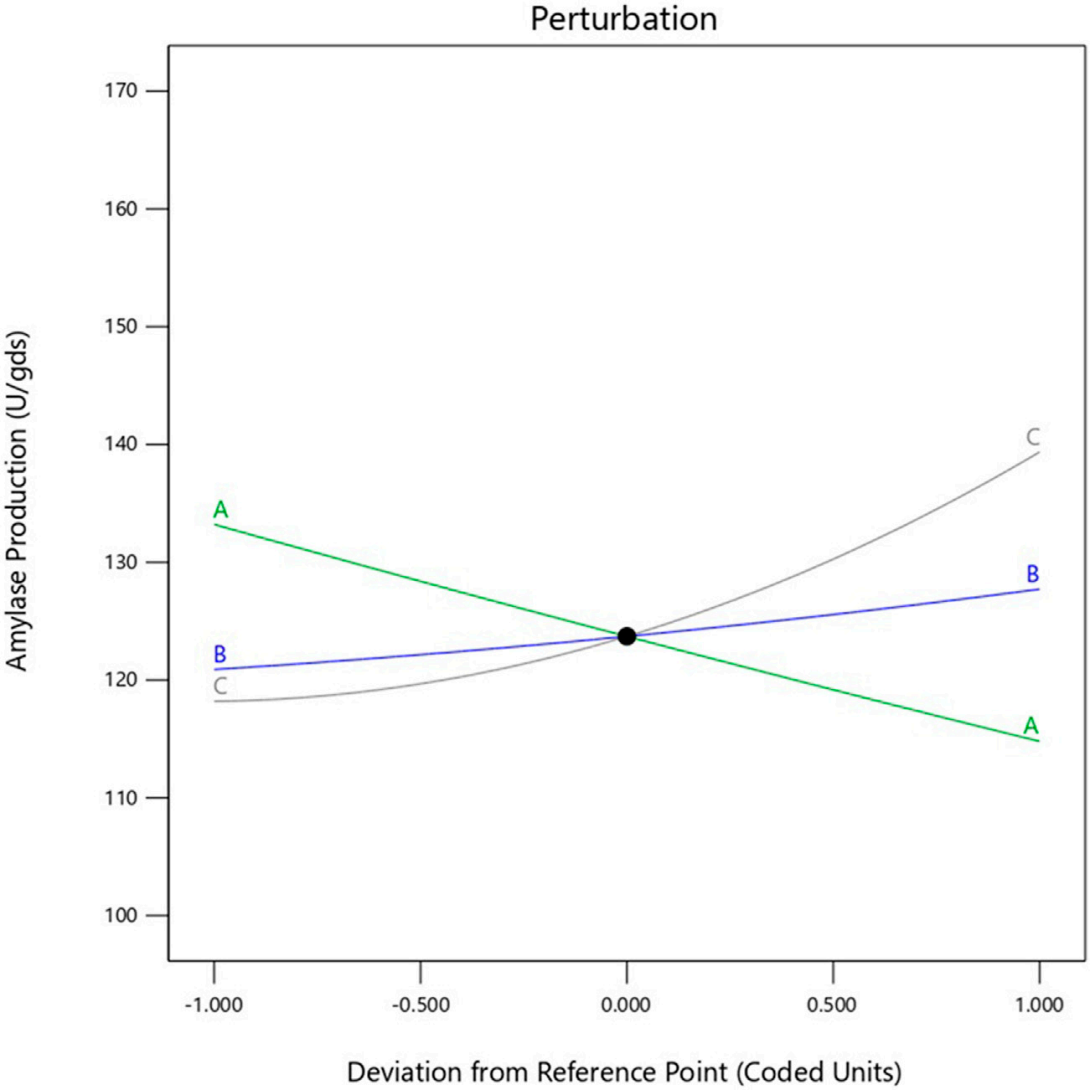
Perturbation plot for α-amylase production.

In the present study, the interaction effects of the three selected media components at different levels on α-amylase yield revealed three different response surface plots. Figure 2a shows α-amylase production as the main effect as well as the interaction and squared effects (non-linear) of starch and yeast extract at different levels; at lower and higher concentrations as per the design matrix in the SSF experiments, both components do not reveal any positive interaction effect on enzyme yield. Increasing the starch concentration does not enhance enzyme yield, whereas a gradual increase in the concentration of yeast extract used resulted in gradual increment of the enzyme yield. Figure 2b illustrates the interaction between starch and calcium chloride when the concentration of yeast extract used was fixed; both starch and CaCl_2_ at the specified lower concentrations did not enhance enzyme yields; further, maintaining CaCl_2_ at the lower concentration and increasing starch to a higher concentration did not improve the yield as well. The calcium chloride concentration in the production medium has a projecting effect on α-amylase yield, and increasing this concentration leads to concomitant increase in enzyme yield; it was observed that higher CaCl_2_ and lower starch concentrations have profound effects on enzyme yield. The interaction of yeast extract and calcium chloride showed a positive effect on enzyme yield, which can also be predicted from the shape of the curve shown in Figure 2c. This increment in enzyme yield was observed with synchronized increments in both components, i.e., yeast extract and CaCl_2_, while both components at their lower concentrations did not show any significant increases in α-amylase yield. The overall interaction effects among the chosen variables indicate that maintaining starch concentration low while concomitantly increasing the concentrations of yeast extract and CaCl_2_ improves enzyme yield. This is clearly illustrated in the perturbation plot shown in Figure 5. The meticulously performed validation experiments corroborate the quadratic model and predicted values of the selected variables.

The enzyme α-amylase is known to reduce HAuCl_4_ salt from Au^3+^ to Au^0^ (Au-NPs) (Rangnekar et al., 2007). The amylase structure analysis revealed that the enzyme has free and exposed S–H groups in its native form and is thus suitable for the reduction of HAuCl_4_ to generate Au-NPs (Kalishwaralal et al., 2009; Rangnekar et al., 2007). Nanoparticles and nanostructures have garnered significant interest owing to their appealing material properties and diverse applications in various fields involving optical devices, sensors, photocatalysis, remediation of organic pollutants, and antibacterial coatings (Ahmad et al., 2015). The ability of *B. subtilis* VSP4 to synthesize Au-NPs was assessed by growing it in an NB as a general cultivation medium; then, the supernatant obtained after 24 h of incubation was used for the green synthesis of Au-NPs. The culture supernatant often has a considerable impact on the synthesis of Au-NPs but requires a longer process time. RSM was implemented to optimize the production medium to enhance amylase production using *B. subtilis* VSP4 under SSF. The generated crude supernatant has sufficient amylase content and can be used to synthesize Au-NPs in a shorter period of time (24 h).

The present work shows that α-amylase production by *B*. *subtilis* VSP4 under SSF could be enhanced by adopting the statistical experimental methodology for efficient synthesis of Au-NPs. The chosen statistical method has notable advantages as it reduces the total experimentation time as well as cost of the media, which directly justify the fermentation economics and provide the best outcomes over the conventional methodology. The α-amylase of *B. subtilis* VSP4 can be used for rapid biosynthesis of Au-NPs having an average size of 5.17 ± 0.85 nm and a surface plasmon resonance peak at 528 nm to cater to various industrial demands.

## Data Availability

The datasets presented in this study can be found in online repositories. The names of the repository/repositories and accession number(s) can be found in the article/supplementary material.

## Abbreviations

SSF, solid-state fermentation; RSM, response surface methodology; CCD, central composite design; PBD, Plackett–Burman design; U/gds, units/gram dry substrate; OD, optical density; OFAT, one factor at a time; TEM, transmission electron microscopy; NB, nutrient broth; Au-NPs, gold nanoparticles; CV, coefficient of variation.
